# Adhesive Capsulitis of the Shoulder. Is there Consensus Regarding the Treatment? A Comprehensive Review

**DOI:** 10.2174/1874325001711010065

**Published:** 2017-02-28

**Authors:** Dimitrios Georgiannos, George Markopoulos, Eirini Devetzi, Ilias Bisbinas

**Affiliations:** 1 Orthopaedics and Trauma Surgery 424 Military General Hospital, Thessaloniki, Greece; 2 Rheumatology 424 Military General Hospital, Thessaloniki, Greece

**Keywords:** Adhesive capsulitis, Shoulder, Treatment

## Abstract

**Background:**

Adhesive capsulitis of the shoulder (ACS) is a common self-limiting condition characterized by disabling pain and restricted movements. Its pathophysiology is poorly understood, clinically it is characterized by stages of pain and stiffness, and finally often patients never recover fully. However, there is no consensus about available methods of treatment for ACS. The aims of this paper are to discuss and develop issues regarding approaches to management in ACS in the stages of it.

**Methods:**

A review of the literature was performed and guidelines for the treatment of that clinical entity for doctors and health care professionals are provided.

**Results:**

Anti-inflammatory medications, steroid and/or hyaluronate injections and physiotherapy is the mainstay of conservative management either alone in the first stages or in combination with other treatment modalities in the later stages. Next line of treatment, involving minor to moderate intervention, includes suprascapular nerve block, distension arthrography and manipulation under anaesthesia. In order to avoid complications of “blind intervention”, arthroscopic capsular release is gradually more commonly applied, and in recalcitrant severe cases open release is a useful option.

**Conclusion:**

Various modalities of conservative management and gradually more surgical release are applied. However, often clinicians choose on personal experience and training rather than on published evidence.

## INTRODUCTION

Adhesive capsulitis of the shoulder (ACS) is a common disease characterized by disabling pain and restricted movements. Neviaser in 1945 suggested that the term “adhesive capsulitis” better described the pathology- adhesions between the axillary fold and the humeral neck and the “misnomer frozen shoulder should be deleted from the medical literature” [1]. The term “Frozen Shoulder” (FS) is coined to Codman in 1934 [2], although it was Duplay in 1896 who described a clinical condition with the same characteristics and named it as “periarthrite scapulohumerale” [3]. The condition has also been termed “frozen shoulder syndrome” [4], and “contracture of shoulder” [5]. The multiple terms used to describe the disease shows poor understanding of the pathology and etiology [6]. Bunker proposed an interim term “HGAC” the acronym for “humeroglenoid acromioclavicular” syndrome, which is quite confusing for patients and doctors [7]. The American Shoulder and Elbow Society (ASES) resulted in a consensus definition of ACS as follows: “a condition characterized by functional restriction of both active and passive shoulder motion for which radiographs of the glenohumeral joint are essentially unremarkable” [8].

## EPIDEMIOLOGY - AETIOLOGY - PATHOPHYSIOLOGY

ACS occurs in 2-5% of the general population and up to 20% in diabetic patients [8]. It occurs more commonly in women between 40 and 60 years and in a quarter of the patients the disease is bilateral [8]. ACS has been classified as primary -idiopathic- and secondary - intrinsic, extrinsic and systemic due to predisposing factors [9, 10]. The most frequent predisposing factor leading to ACS is diabetes mellitus with its incidence been in diabetes patients between 10-36% [11]. Further, associations include cardiac disease, pulmonary disease, Parkinson's disease and stroke, hyperthyroidism, hypothyroidism, scleroedema, Dupuytren’s disease or previous non-shoulder surgery including cardiac surgery, thoracic, neck surgery or neurosurgery or shoulder trauma as rotator cuff injury [11].

The pathophysiology of ACS is not clear. It starts without a specific precipitating event, as an inflammatory reaction in the shoulder capsule. The presence of cytokines is evidence of a possible autoimmune process, which however has not established relations or aetiology [12]. After active fibroblastic proliferation in the shoulder capsule, fibroblasts start transforming to myofibroblasts causing inflammatory contracture, capsular hyperplasia and fibrosis reducing the capsular volume and restricting the glenohumeral movements [13].

Pain and decreased range of motion (ROM) are the principle symptoms all the way through the course of adhesive capsulitis. The symptoms can persist for 12-24 months. Interestingly, 10% of patients never recover full ROM. However, this loss of motion is seldom functionally limiting [12].

## STAGES AND DIAGNOSTIC CRITERIA

It is commonly believed that ACS is an idiopathic, self-limiting syndrome divided into four consecutive stages lasting approximately 24 months in total [6]; 1. Painful stage: characterized by gradual onset of symptoms which persist for less than 3 months and consist of mild to severe pain, mild limitation of range ROM, and inability lying on the affected shoulder. 2. Freezing stage: symptoms continue for 3-9 months, characterized by severe nocturnal pain and significant loss of both active and passive ROM. 3. Frozen stage: symptoms persist for 9-14 months and characterized by shoulder stiffness and pain at the end of motion or at night. 4. Thawing stage: occurs between 15-24 months and characterized by minimal pain and a gradual improvement of ROM due to capsular remodeling.

The formal diagnostic criteria include: 1.painful and stiff shoulder for at least 4 weeks; 2. severe shoulder pain that interferes with activities of daily living or work; 3. night pain; 4. painful restriction of both passive and active shoulder range of motion (elevation <100°, external rotation >50% restriction); and 5.normal radiological appearance [14]. Aim of this article is to review the current literature on treatment of this painful, disabling and often debilitating condition.

## TREATMENT

Some authors believe that pain relief is the main objective of all treatments for ACS [15]. However, considering the functional disability and its impact on the patients, this objective should be refined to early pain relief and functional restoration [16]. Choice of treatment approach depends upon the patient’s functional status at the time of presentation. Generally speaking a trial of conservative care is warranted for 6 months provided the clinician closely monitors progress. If progress stalls or the condition worsens, an alternate approach to treatment should be considered [17].

### Conservative Treatment

#### Oral Medication

Non steroidal anti-inflammatory drugs (NSAIDs) have been widely used for the treatment of ACS, especially at the painful first phase of disease. According to the literature, some studies- even with questionable clinical evidence- showed significant improvement of pain in patients treated with NSAIDs compared with placebo [18]. Others support the use of NSAIDs at the earlier inflammatory stages of the ACS claiming that they may provide short-term pain relief [19, 20].

Oral corticosteroids can also be used and have been shown to improve pain, especially night pain [18], and ROM in the short term [21-25]. Buchbinder *et al.* [24] in a randomized controlled trial (RCT) concluded that oral steroids provide significant short term benefits in pain relief, ROM and function. A recent systematic review [25] concluded similar results, with “silver” level evidence that oral steroids provide significant short-term improvement but the effect may not be maintained beyond six weeks. Minimal adverse effects were reported with the use of steroids.

#### Intra-Articular Injection

Intra-articular corticosteroid injections are used commonly to manage ACS for many years, quelling the inflammation process and reducing the patient’s symptoms [26]. The number of injections as well as the injection techniques used varies. The dose of steroid used ranges from 20 to 60 mg Triamcinolon [27, 28]. Often corticosteroid injections are performed as a mixture with a local anesthetic. The improvement of ROM is attributed to the effect of the local anaesthetic medication that reduces the pain and the muscle spasm [29]. The effect has been described to last up to 6 weeks, and the injections are more effective when synovitis is present during the early stage of ACS [30, 31]. The injection can be targeted into the glenohumeral or subacromial space without significant differences as about its effectiveness [32]. Multiple studies have shown that the use of ultrasound or fluoroscopy to guide injections improves the accuracy of these injections compared with a blind technique [33-36]. Juel *et al.* [37] has recently described a new injection technique with an ultrasound-guided injection of 20 mg triamcinolon into the rotator interval with significant effects on ROM after 12 weeks. Steroid injections were compared with placebo, supervised physiotherapy and intra-articular sodium hyaluronate injection in three high quality RCTs [38-40].

Sodium hyaluronate injection into the shoulder joint is a relatively new intervention for the management of adhesive capsulitis. Hyaluronate is a major component of connective tissue and has metabolic effects on articular cartilage, synovial tissue and synovial fluid [41]. Hyaluronic acid injections have been shown to be beneficial for persistent shoulder pain especially when the main reason is osteoarthritis of the glenohumeral joint [42, 43]. Harris *et al.* in a review article showed that hyaluronate injection into the glenohumeral joint is a safe and effective procedure improving pain and ROM at short-term follow up in patients with ACS [44]. However, Callis *et al.* in a comparative study demonstrated that hyaluronic acid intra-articular injections were not as effective as the intra-articular corticosteroid injections or physiotherapy for the treatment of ACS [38]. Finally, Lin-Fen Hsieh *et al.* in a RCT showed that the addition of hyaluronic acid injections to conventional physiotherapy did not produce a significant added benefit for the treatment of patients with ACS and may increase unnecessary medical expenditures [45].

#### Physiotherapy

Physiotherapy is considered an important factor of maintaining movement by preventing capsular contraction and regaining ROM once symptoms allow and it is widely accepted that it should be used in the conservative management of ACS [46, 47]. A variety of interventions were used, which included among others heat or ice applications, active and passive ROM exercises, mobilization techniques, stretching, patient’s education and supervised home exercises, laser therapy, transcutaneous electrical nerve stimulation and proprioceptive neuromuscular fascilitation techniques (PNF) [48]. The use of ultrasound, massage, iontophoresis and photophoresis has been discouraged by some authors and they seemed to be of no benefit for the treatment of ACS, reducing the likelihood of a favorable outcome [49].

Joint mobilization techniques such as traction and glide were used to stretch the adhered capsule and improve the physiologic accessory movements [50]. Traction involved distraction of one articular surface perpendicular to the other and gliding involved translational movement of one articular surface parallel to the other. Johnson *et al.*, in a study comparing anterior versus posterior glide mobilization, concluded that posterior joint mobilization was more effective than anterior for improving external rotation after three therapeutic sessions [51]. The intensity of stretching has to match with the patient’s patience and the tissue’s ability to cope with physical stress [52].

Diercks *et al.* in an RCT of 77 patients showed that intensive physical rehabilitation can be counter-productive comparing one group performing passive stretching and mobilization beyond pain limits (intensive physiotherapy group) with a second group performing active and actively assisted exercises within pain limits (supervised neglect group). At two years only 63% of the first group and 89% of the second group had good shoulder function [53]. However, Vermeulen *et al.* in a RCT concluded that high grade mobilization techniques are more effective than low grade ones in the management of ACS of the shoulder despite of patients were improved significantly with both treatment strategies [48]. Another recent study by Russel *et al.* showed that anxiety and depression appear to be an important part of ACS symptoms and physiotherapy interventions in a group exercise class provided superior outcomes in relieving signs and symptoms because they particularly addressed this aspect of the condition [54]. Finally, when selecting a physical treatment method for ACS it is important to consider the patient’s symptoms, stage of condition and recognition of different patterns of motion loss [55]. It is also important to educate the patient explaining the pathology and self-limiting character of the condition, acknowledging that full ROM might never be restored [52].

#### Suprascapular Nerve Block

Suprascapular nerve block (SNB), first described in 1941, aims to block the nerves to the glenohumeral joint as they branch from the suprascapular nerve near the scapular notch [56]. The block could be repeated twice weekly for a total of 2-4 treatments [57]. Jones *et al.* compared the therapeutic effects of SNB to intra-articular corticosteroid injection, and found that the technique was safe, effective and has several benefits over intra-articular injection [58]. A RCT conducted by Dahan *et al.* compared three SNB with bupivacaine at weekly interval with placebo. SNB offered significant pain relief but no improvement with regard to ROM [59]. Ultrasound-guided placement of a perineural catheter to provide continuous SNB has also been proposed in a case report [60].

#### Distension Arthrography

Distension arthrography is a procedure where an injection of a saline solution and/or steroids or air into the shoulder joint breaks up the adhesions that might limiting the movements of the glenohumeral joint and causing pain and disability. Arthrographic distension of the shoulder capsule leading to capsular rupture was first described by Anderson and Lundberg [61]. Distension could be done with a mixture of long acting anaesthetics, steroids, saline or air. Because of the inherent compressibility of air, distension of a contracted capsule is more difficult than if saline is used [62]. Dependent upon the contracted state of the joint capsule, distension usually occurred when between 10ml and 55ml of normal saline had been injected. Rupture usually occurred through the subscapularis bursa and occasionally down the biceps sheath [62]. Corbeil *et al.* contacted a double-blind prospective trial comparing intra-articular steroid injection vs arthrographic distension and found no significant difference between the two groups [63]. Jacob *et al.* compared three interventions in a RCT. Shoulder distension with air, intra-articular steroid injection and distension with air and steroids. They concluded that intra-articular steroid injection and distension plus steroids to be superior to distension alone, but there was no significant difference between steroid injection with or without [64]. Buchbinder *et al.* compared arthrographic distension with steroid and saline versus placebo. There was significant improvement of ROM, pain and disability in the distension group compared to placebo group [24]. A RCT conducted by Gam *et al.* showed significant improvement in ROM and lower need for analgesics in the arthrographic group, but no difference in pain or function compared with the steroid injection group [65]. Hsu and Chan reported a prospective study comparing MUA and physiotherapy with arthroscopic distension and physiotherapy, and with physiotherapy alone. They reported no significant difference between the first two groups regarding pain, ROM and function [66]. Other studies although non RCTs, but with long follow-up (up to 28 months) concluded that arthrographic distension is a safe and effective treatment of frozen shoulder that gives long-term improvement [62, 67, 68]. Park *et al.* in a RCT, compared ultrasound guided capsular distension with hyaluronic acid with steroid injection alone [69]. They concluded that capsular distension with hyaluronic acid was as effective as the steroid injection alone regarding the pain relief and function but more effective regarding the passive external rotation movements [69].

#### Manipulation Under Anaesthesia (MUA)

MUA can be considered after 6 months of refractory pain and stiffness in patients with ACS [19, 70]. It consists of a controlled, forced restoration of shoulder movements. Elevation and abduction releases the inferior capsule due to rupture of the capsule from the humeral neck. Forced external rotation tears the coracohumeral ligament allowing for improvement of rotational movements [46]. Dodenhoff *et al.* concluded that MUA is a safe procedure that provides early improvement of the shoulder function [16]. Farrell *et al.* reported excellent results in 70% of patients treated with MUA at 15 years follow-up [71]. Wang *et al.* concluded that MUA accelerates ACS recovery and improves shoulder function and symptoms within a short period of time. They were also reported neither recurrence of symptoms in 8 year follow-up nor residual complications [72]. Jacobs *et al.* conducted a RCT comparing MUA versus intra-articular steroids and concluded that there is no difference regarding the speed of reduction in pain or improvement in function [73]. Kivimaki *et al.* reported no differences at 3, 6 and 12 months between MUA and exercises compared with physiotherapy exercises alone in an RCT [74]. DeCarli *et al.* showed that patients undergoing a MUA with an arthroscopic capsular release had better outcomes at 6 weeks but no difference at 12 weeks compared with patients undergoing intra-articular corticosteroid injection [75]. Reichmister andFriedman reported that 97% of patients treated with MUA had significant improvement of pain and ROM, however 8% required a second MUA [76].

### Surgical Treatment

#### Arthroscopic Release

The effectiveness of arthroscopy in treatment of ACS has been confirmed by several studies [77-80]. Ogilvie-Harris *et al.* first described the arthroscopic anterior capsulotomy for the treatment of ACS. They suggested that the inferior capsular release should be avoided in order to prevent injury to the axillary nerve [81]. Le Lievre *et al.* [82] showed that patients underwent arthroscopic capsular release maintained the initial good results regarding pain, function and ROM for over 7 years [82]. Interestingly, improvement of internal rotation might be very poor. Several authors performed a posterior release to increase the range of internal rotation however this has shown to make no difference [83]. There is no consensus regarding the extent of capsular release and which structures should be involved in release. Many authors release only the rotator cuff interval and the contracted coracohumeral ligament claiming excellent results [84-86] while some others suggest that releasing the superior edge of subscapularis is essential t restore the external rotation [87-89]. A posterior capsular release might improve a severely restricted internal rotation [13, 83]. A ‘360° capsulectomy’ has been recently utilized by several surgeons although this technique associated with higher risk of axillary nerve injury [19, 89-91]. Chen *et al.* reported poor functional outcomes at 3 months after capsulotomy without inferior release [92]. Ogilvie-Harris *et al.* compared arthroscopic release with MUA. They concluded that arthroscopic release is the treatment of choice for resistant ACS ensuring more substantial and rapid ROM and pain improvement controlled with lower risk of complications [93].

#### Open Release

With the development of arthroscopic techniques, open release is nowadays rarely performed and is mentioned only for historical purposes. There are several studies in the literature suggesting that open release could be used for recalcitrant cases of ACS and good results with complete pain relief and full ROM restoration are reported [94, 95].

## DISCUSSION

The level of evidence of studies regarding the various treatments for ACS is generally low especially with regard to surgical treatments. RCTs are difficult to perform because of the difficulties regarding the clinical diagnosis as well as the various stages of ACS in the recruited patients. Therefore, most of the studies are mainly case series or retrospective comparative studies [19].

Despite the widespread use of NSAIDs, there is no evidence in the literature to support their effectiveness for the treatment of ACS [18-20]. Oral steroids appear to provide significant short-term improvement but their effect may not be maintained beyond six weeks. Furthermore the use of oral steroids for a long period involves long-term systematic side effects [24, 25].

Although, many studies showed significant early benefit of intra-articular injections on pain and shoulder ROM, no differences were found after three months between various treatments. They may be more efficacious in the early inflammatory stage without substantial capsular contracture however this finding has yet to be proven with higher level studies [27, 28]. The combination of steroid injection with physiotherapy appeared more effective than physiotherapy or steroid injections alone in the recovery of range of motion [47].

Physiotherapy and exercises are often used to prevent capsular contracture and improve shoulder ROM. Home exercise program with gentle stretching seemed to provide better results than more intensive physiotherapy regimes although there is no sufficient evidence about the most effective physiotherapy [96, 97]. Regarding the physical therapy, there is good evidence that the laser therapy and the deep heating especially is very effective if applied additionally to mobilization techniques or exercise programs [98].

Suprascapular nerve block is proved a safe, simple, highly successful and reproducible alternate for the treatment of ACS in the primary care [58, 99]. It could be used alone or in combination with steroids for additional pain control. Blockage of the suprascapular nerve, which contains a substantial proportion of sympathetic fibers supplying the shoulder joint, cause significant pain relief [57].

Capsular distension of shoulder joint has been recommended for patients with ACS resistant to conservative treatment [24, 62, 67]. There is only “silver” level evidence that arthrographic distension with saline and steroid provides short-term benefits in pain, ROM and function in ACS and that no conclusions from the existing RCT’s can be made on the long-term outcome [25].

There is no consensus about the safety of MUA. Several iatrogenic complications, such as haemarthrosis, glenohumeral ligament rupture, glenoid labral detachment, SLAP lesions, rotator cuff tendon tears, and humeral fractures have been reported [100]. The use of injections is recommended rather than MUA and physiotherapy as a first-line treatment for patients in the freezing phase of ACS [73].

Arthroscopic capsular release has been favored over MUA as it allows a complete release of the contracted capsule, reduces the chance of fracture and provides immediate improvement [82]. However, there is still controversy regarding the optimum way of release. The primary source of pathology in ACS is the rotator interval [9]. Therefore, it is the release of that area in conjunction with the inferior capsule that provides the greatest improvement in ROM [83, 65]. In contrast, several authors have recently utilized the ‘360° total capsular release’ [89-91]. The major advantage of arthroscopic capsular release compared to open one, is that the physical therapy and exercise can be initiated immediately after surgery [89]. It is also valuable in patients with osteoporosis that are difficult and dangerous to perform MUA. Other benefits of the arthroscopic capsular release include the ability to allow release of the rotator interval, the anterior and posterior capsule, and the inferior pouch; debridement of the subscapularis tendon; denervation of the glenohumeral joint capsule; removal of synovial tissue [89].

Open release is considered the last chance for the most recalcitrant cases after failure of the arthroscopic release and MUA as associated with several complications such as prolonged recovery, stiffness, postoperative pain that can delay early mobilization [47, 94, 95].

Obviously, there is insufficient evidence to draw solid conclusions about which treatment is the most effective [101]. In the absence of clear direction from the literature a realistic approach to patient management needs to be planned. A survey study [102] among healthcare professionals (orthopaedic surgeons, general practitioners, physiotherapists) stated that most professionals recommended non-operative treatment in the painful phase including oral analgesia and physiotherapy. 18% of the professionals recommended injections in the first instance, 3% recommended arthroscopic release and 1.4% advocated capsular distension. Another survey study among shoulder orthopaedic surgeons enquiring on surgeons’ treatment preference revealed that the management of ACS varies both with regard to non-surgical and surgical options. Most of them based their choice of treatment on personal experience and training rather than on published studies [103].

The treatment of ACS should be based on the stage of the disease and on patient characteristics. A treatment algorithm is proposed in Table **1**. Well educated patients and reassurance regarding the disease increases compliance and improves the treatment outcome. However, it is important to emphasize that the full ROM may never recover. *In the painful phase*, the goal of treatment is to reduce pain and to preserve shoulder mobility and function. Oral NSAIDs and/or intra-articular steroid injections or intra-articular hyaluronate injections indicated for the first 4 weeks. Additionally, stretching and exercise program within the pain threshold could be used for better results. *In the freezing phase*, the goal of treatment is to prevent stiffness and reduce the formation of intra-articular adhesions. Physiotherapy plays the most important role of treatment in the freezing phase. The exercise program includes passive mobilization, active exercises, daily home exercises and internal-external stretching exercises with the use of pulleys. The duration and intensity of program should be based on the pain threshold. Intra-articular corticosteroids are generally used to improve the synovitis, thus reducing the natural history of disease. Capsular distension is a treatment option, very favorite among the shoulder surgeons in this phase. *In the frozen phase*, the goal of treatment is to improve the marked loss of ROM of the shoulder. Capsular distension, MUA, and arthroscopic release could be used alone or in combination to restore a proper dynamic shoulder joint. Physiotherapy is used to maintain the achievements of the invasive technique used. The control of pain with NSAIDs or injections is important to increase the intensity, duration and frequency of exercises. The addition of deep heating to stretching exercises and mobilization can produce a greater improvement in ROM through the relaxation of the surrounding musculature. *In the thawing phase*, the goal of treatment is the restoration of the normal function of the shoulder. Arthroscopic release is widely used. Open release could be used for very stiff shoulders. Additionally, stretching and proprioceptive exercises are prescribed in order to correct the compensatory movements and restore a well-built dynamic shoulder joint.

## CONCLUSION

Adhesive capsulitis of the shoulder is a disabling disease often with up to 24 months recovery. Although, many treatment modalities have been reported for the management of the ACS, there is still no consensus in the literature regarding which therapeutic option is superior, mostly because of a lack of high-level evidence. Given that ACS is a self-limiting condition, conservative management is the first choice in the first stage of the disease. Physiotherapy and active and passive exercises, combined with oral medication and/or intra-articular corticosteroid injections, are considered most important in the conservative treatment of ACS. After failure of conservative treatment invasive options can be considered. It seems that distension arthrography combined with gentle MUA is being increasingly used as a first line of invasive treatment and is being considered more and more by clinicians before arthroscopic capsular release. High quality RCTs with long follow-up should be designed to compare different treatment options and to formulate precise guidelines about the treatment of ACS.

## Figures and Tables

**Table 1:** Goals and treatment modalities which are suggested-applied for ACS treatment in each phase.

**ACS Treatment**	**Goal**	**Treatment Modality**
**Painful phase**	pain reduction and preservation of function	NSAIDs - i.a. steroid injections i.a. hyaluronate injections physiotherapy / hydrotherapy
**Freezing phase**	prevention of adhesion formation	NSAIDs - i.a. steroid injections capsular distension treatment physiotherapy
**Frozen phase**	ROM improvement	NSAIDs - i.a. steroid injections capsular distension treatment MUA arthroscopic release physiotherapy
**Thawing phase**	restoration of joint normal function	arthroscopic release open release physiotherapy

## LIST OF ABBREVIATIONS

ACS: = Adhesive capsulitis of the shoulder
ASES: = American shoulder and elbow society
FS: = Frozen shoulder
HGAC syndrome: = Humeroglenoid acromioclavicular” syndrome
MUA: = Manipulation under anaesthesia
NSAIDs: = Non-steroidal anti-inflammatory drugs
PNF: = Proprioceptive neuromuscular fascilitation techniques
RCT: = Randomized controlled trial
ROM: = Range of motion
SNB: = Suprascapular nerve block
